# Omission of dexamethasone in paclitaxel premedication regimens: protocol of the multicentre, randomised, non-inferiority DEXASTOP trial

**DOI:** 10.1136/bmjopen-2025-102770

**Published:** 2025-04-25

**Authors:** Michiel Zietse, Luuk C Aalders, Leontine E A M M Spierings, Nikki De Rouw, Wouter Dercksen, Virgil A S H Dalm, Esther Oomen-de Hoop, Frederick W Thielen, Birgit C P Koch, Ron H J Mathijssen, Leni van Doorn, Roelof W F van Leeuwen

**Affiliations:** 1 Department of Hospital Pharmacy, Erasmus MC Universitair Medisch Centrum Rotterdam, Rotterdam, Netherlands; 2 Department of Internal Medicine, Alrijne Hospital, Leiderdorp, Netherlands; 3 Department of Clinical Pharmacy, Amphia Hospital site Molengracht, Breda, Netherlands; 4 Department of Internal Medicine, Maxima Medical Centre, Veldhoven, Netherlands; 5 Department of Immunology, Erasmus University Medical Center, Rotterdam, Netherlands; 6 Department of Internal Medicine, Division of Allergy & Clinical Immunology, Erasmus University Medical Center, Rotterdam, Netherlands; 7 Department of Internal Medicine, Erasmus MC Cancer Center, Rotterdam, Netherlands; 8 Department of Health Technology Assessment, Erasmus Universiteit Rotterdam Erasmus School of Health Policy and Management, Rotterdam, Netherlands; 9 Erasmus Centre for Health Economics Rotterdam, Erasmus University Rotterdam, Rotterdam, Netherlands; 10 Department of Medical Oncology, Erasmus MC Cancer Institute, Rotterdam, Netherlands

**Keywords:** ONCOLOGY, CHEMOTHERAPY, Quality of Life, CLINICAL PHARMACOLOGY

## Abstract

**Introduction:**

Standard premedication for paclitaxel-based chemotherapy includes dexamethasone and an histamine 1-antagonist to prevent hypersensitivity reactions (HSRs). However, the pharmacological rationale for dexamethasone is limited, and its use is associated with adverse effects such as hyperglycaemia, insomnia and immunodeficiency, negatively impacting health-related quality of life (HRQoL). No clear link has been established between dexamethasone dose, administration route and HSR incidence. Previous studies suggest that discontinuing dexamethasone beyond the second administration does not increase HSR risk. Despite this, dexamethasone remains standard practice. This trial evaluates whether complete omission of dexamethasone as paclitaxel premedication is non-inferior to the standard regimen in preventing clinically relevant HSRs (Common Terminology Criteria for Adverse Events (CTCAE) grade≥3).

**Methods:**

The DEXASTOP trial is a prospective, multicentre, randomised, non-inferiority study conducted in four hospitals in the Netherlands. 500 adult patients receiving paclitaxel-based chemotherapy for any solid tumour indication will be randomised 1:1 to receive either standard premedication with dexamethasone or an experimental regimen without dexamethasone for up to five paclitaxel administrations. The primary endpoint is the incidence of clinically relevant HSRs (CTCAE grade≥3). Secondary endpoints include the incidence and severity of all-grade HSRs, the number of paclitaxel administrations before the first HSR, dexamethasone-related adverse events, HRQoL and cost-effectiveness from a healthcare and societal perspective.

**Ethics and dissemination:**

This study has been approved by the Erasmus Medical Centre Ethics Committee (reference MEC-2024-0030, protocol version 3, May 2024). Study findings will be published open access in peer-reviewed journals and presented at national and international meetings. Results will be shared with patients, healthcare professionals and the public. Positive outcomes will be implemented in clinical practice, and trial data will be submitted to the EU Clinical Trials Information System for public access.

**Trial registration number:**

NCT06118710.

**STRENGTHS AND LIMITATIONS OF THIS STUDY**

- First multicentre, randomised, non-inferiority trial assessing complete omission of dexamethasone in paclitaxel premedication.
- Evaluation of multiple secondary outcomes, including health-related quality of life, cost-effectiveness and dexamethasone-related adverse events.
- Inclusion of patients with different cancer types, ensuring broad applicability.
- Not blinded, which may introduce some bias in patient-reported outcomes.

## Introduction

 Hypersensitivity reactions (HSRs) are immunological responses to a drug that lead to adverse reactions and are frequently observed as side effects during paclitaxel infusion. These HSRs range in severity from mild erythematous rashes to life-threatening anaphylaxis.^1^ The exact mechanism underlying paclitaxel-induced HSRs remains unclear, but Cremophor EL, the solubilising agent in paclitaxel formulations, is believed to play a key role.^1^

HSRs most commonly occur within the first 10 min of paclitaxel infusion, typically during the first or second treatment cycle, with prior studies reporting that approximately 97% of reactions occur within this timeframe.^2 3^ Before the introduction of premedication regimens, HSRs (all grades) were seen in 25–42% of all patients using paclitaxel.^4 5^ With the implementation of standard premedication, including a histamine 1 (H1)-antagonist (eg, cetirizine) and dexamethasone, the incidence of HSRs (all grades) has decreased to approximately 20%.^2 6 7^

Although dexamethasone is routinely given to prevent paclitaxel-induced HSRs, its added value has never been thoroughly studied since paclitaxel’s registration in 1993.^4^ Moreover, the pharmacological rationale for administering dexamethasone 30 min before paclitaxel infusion is questionable as its biological activity typically requires several hours to take effect.^8^ Furthermore, it has been suggested that paclitaxel-induced HSRs are not allergenic, but instead mediated via direct histamine release through non-IgE-mediated mast cell degranulation.^9^,^11^

Several studies have indicated that discontinuing dexamethasone after the second paclitaxel administration does not increase the risk of HSRs.^12^,^16^ Additionally, lower doses of dexamethasone in premedication regimens have been shown to be feasible,^17^ and there seems to be no statistically significant association between the dose or the administration route (intravenous or oral) and the HSR rate.^18^ Despite its widespread use, dexamethasone is associated with adverse effects, including hyperglycaemia, immunodeficiency, mood disturbances, sleep disorders and weight gain, all of which negatively impact patients’ health-related quality of life (HRQoL).^19 20^ Therefore, the routine administration of dexamethasone in paclitaxel premedication may expose patients to unnecessary risks without clear clinical benefit. Therefore, discontinuing dexamethasone could improve HRQoL, reduce healthcare utilisation and costs, as well as optimise the premedication regimen.^21 22^

However, head-to-head studies on the added value of dexamethasone in the prevention of paclitaxel-induced HSR have not yet been conducted. Therefore, our study aims to demonstrate that premedication regimen without dexamethasone is non-inferior to the standard of care premedication regimen with dexamethasone, based on the incidence of clinically relevant paclitaxel-induced HSRs (grade≥3 as per the Common Terminology Criteria for Adverse Events (CTCAE) V.5.0).^23^

## Methods and analysis

This protocol summary follows the Standard Protocol Items: Recommendations for Interventional Trials Statement.^24^

### Setting

The DEXASTOP trial is a prospective, randomised, multicentre, non-inferiority study conducted at the Erasmus University Medical Centre (Rotterdam), Amphia Hospital (Breda), Alrijne Hospital (Leiderdorp) and Máxima Medical Centre (Veldhoven).

### Objectives

The *primary objective* is to evaluate the incidence of clinically relevant HSRs (grade≥3 as per CTCAE V. 5.0) during paclitaxel-based chemotherapy with a local standard-of-care premedication regimen with dexamethasone compared with an experimental premedication regimen without dexamethasone.

*Secondary objectives* are as follows:

- To determine the incidence and severity of any grade of HSRs as defined by CTCAE V. 5.0.
- To determine the number of paclitaxel administrations and cumulative dose (mg) until the first HSR occurrence.
- To determine the incidence and severity of adverse events (AEs) related to dexamethasone.
- To determine the effect of dexamethasone on the patient’s quality of life.
- To determine the cost-effectiveness of the premedication regimens with and without dexamethasone from both a healthcare and societal perspective.

### Eligibility criteria

- Age≥18 years.
- Diagnosis of a solid tumour with a planned paclitaxel-based chemotherapy regimen for any indication and at any dose.
- Mastery of the Dutch language.
- Able and willing to provide written informed consent.
- No prior treatment with a paclitaxel-based regimen.
- No indication for paclitaxel in combination with moderately or highly emetogenic chemotherapy requiring dexamethasone as anti-emetic prophylaxis (eg, carboplatin area under the curve (AUC)>4).
- No known hypersensitivity to paclitaxel, carboplatin, cetirizine or excipients (eg, benzyl alcohol).
- No concomitant use of systemic corticosteroids for any indication other than paclitaxel premedication.
- No confirmed and ongoing pregnancy.
- No concurrent participation in an exercise trial.

### Study treatment

**Figure 1:**
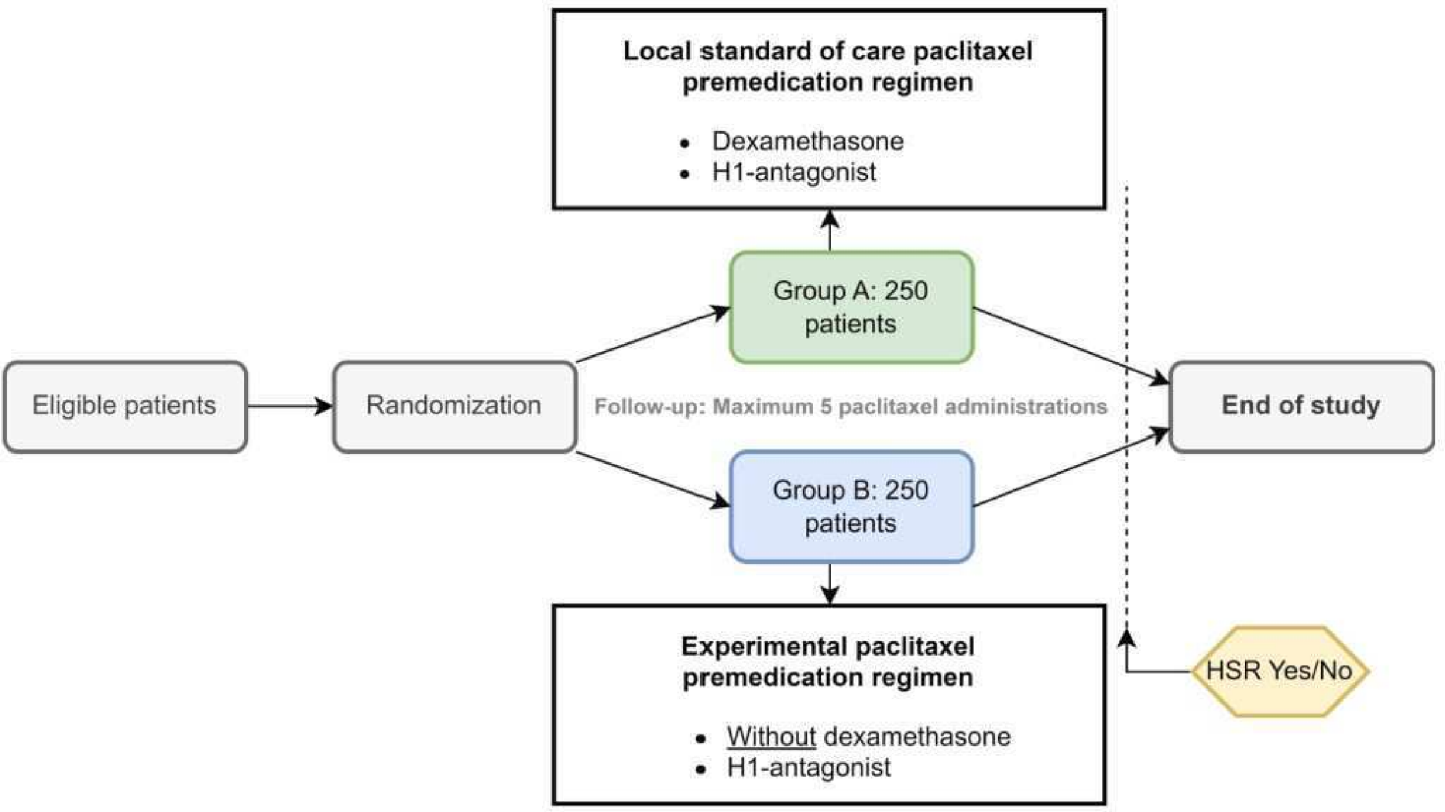
Study design: eligible patients will be randomised (1:1) to receive either the local standard of care paclitaxel premedication regimen (group A) or the experimental regimen without dexamethasone (group B). Hypersensitivity reactions (HSRs) will be assessed over a maximum of five paclitaxel administrations. H1, histamine 1.

The study design is illustrated in figure 1, while the flow chart is provided in online supplemental file 1. Following written informed consent, patients will be randomised in a 1:1 ratio by the investigators via Castor Electronic Data Capture (EDC). Given the possible association between tumour type and incidence of HSRs, tumour type will be included as a stratification factor.^25^

This study compares two premedication regimens for paclitaxel-based chemotherapy: the local standard of care, which includes dexamethasone, and an experimental regimen without dexamethasone. Premedication will be administered 30 min before paclitaxel infusion. Patients in the control group will receive 10 mg cetirizine orally or 2 mg clemastine intravenously, along with dexamethasone (10 mg intravenously or 8 mg orally). Patients in the experimental group will also receive an H1-antagonist but without dexamethasone. Treatment allocation will not be blinded.

Patients are considered evaluable if they receive at least two and up to five paclitaxel infusions or if they experience an HSR during the study. A maximum follow-up of five administrations was selected based on prior evidence, indicating that HSRs rarely occur beyond the fifth infusion.^25^ If a patient experiences an HSR, they will be switched to the standard dexamethasone-based premedication regimen for subsequent infusions and will be considered off-protocol from that point onward.

#### Response evaluation

The oncology medical staff will prospectively assess the grade of HSRs. During and after each paclitaxel infusion, patients will be closely monitored according to standard care procedures. The occurrence of an HSR, as defined by CTCAE V. 5.0 infusion-related reactions, will be documented by research nurses at the end of each paclitaxel administration using standardised case report forms (CRFs).^23^ A grade 3 HSR is defined as a reaction persisting for more than 10 min despite symptomatic treatment or a reaction that reoccurs after initial improvement, while reactions resolving within 10 min are classified as grade 2 (prompt response).

### Sample size

Based on previous studies, it is expected that 1.6% of the patients in both treatment groups will experience HSR (grade≥3).^25^ The non-inferiority margin was set at 4%, meaning the HSR rate in the dexamethasone-free group can be at most 4% higher than in the control group. This margin was determined through literature review, expert consultation and clinical feasibility considerations. Given the variability in reported paclitaxel-induced HSR rates, a 4% margin remains clinically acceptable and is more stringent than margins used in similar studies.^218 25^,^28^ To achieve 80% power at a one-sided significance level of 0.025 using a binomial test, 448 patients (224 per group) are required. Accounting for an estimated 10% dropout rate, a total of 500 patients (250 per group) will be included.

### Statistical analyses

The primary analysis will follow a per-protocol (PP) approach, which is more appropriate and conservative for a non-inferiority trial. The PP population includes patients who received the assigned premedication, completed at least two and up to five paclitaxel infusions (unless an HSR occurred earlier) and had no major protocol violations affecting treatment exposure. A modified intention-to-treat analysis, including all randomised patients who received at least one paclitaxel infusion, will be conducted as a sensitivity analysis.

A two-tailed p value<0.05 will be considered statistically significant. P values for secondary endpoints will be descriptive and hypothesis-generating. No imputation for missing data will be performed. Categorical variables will be presented as n (%) and compared using the Mantel-Haenszel test, stratified by randomisation factors. Continuous variables will be reported as mean±SD or median (IQR), depending on distribution, and compared using unpaired t-tests or Wilcoxon/Mann-Whitney U tests, as appropriate.

#### Analysis of primary study parameter(s)

The primary endpoint is the incidence of clinically relevant HSRs, defined as an immunological response to paclitaxel resulting in an adverse reaction of grade≥3 (per CTCAE V. 5.0). Non-inferiority will be assessed by evaluating whether the upper bound of the two-sided 95% CI for the difference in HSR rates exceeds 4%. The Mantel-Haenszel test, stratified by randomisation factors, will be used to compare the proportion of patients experiencing an HSR grade≥3 between treatment arms. A logistic regression model will adjust for stratification factors and assess the robustness of findings.

#### Analysis of the secondary endpoints

- The incidence of HSRs (all grades, per CTCAE V. 5.0) will be reported as absolute numbers and percentages per treatment arm. Comparisons between groups will be performed using the Mantel-Haenszel test, adjusting for stratification factors. The severity of HSRs (grade distribution) will be compared using the χ2 test for trends.
- The number of paclitaxel administrations and cumulative dose (mg) until the first HSR occurrence will be summarised using descriptive statistics.
- HRQoL will be assessed using the European Organisation for Research and Treatment of Cancer Quality of Life Questionnaire Core 30 (EORTC QLQ-C30) and EuroQol five-dimension five-level questionnaire (EQ-5D-5L) at baseline and the end of study. Scale scores (0–100) for the QLQ-C30 will be calculated according to the EORTC scoring manual, while EQ-5D-5L utility values will be computed using the Dutch valuation set.^29^ Differences between treatment arms will be analysed using analysis of covariance, adjusting for baseline values.
- Symptoms associated with dexamethasone will be evaluated using the Dexamethasone Symptom Questionnaire.^19^ The total scores will be described per measurement in time (ie, at baseline and after each paclitaxel infusion). A linear mixed model will be used to compare the scores over time between the groups.
- Healthcare and societal costs will be assessed using the iMTA Medical Consumption Questionnaire and iMTA Productivity Cost Questionnaire. Direct healthcare costs (medication, inpatient and outpatient resource use) and societal costs (travel expenses, productivity losses, informal care) will be included in the analysis. Mean costs per paclitaxel administration (with and without dexamethasone) will be reported in euros as mean and SD, reflecting the expected skewness of cost data. The Mann-Whitney U test will be used to compare cost differences between treatment groups.

### Recruitment

This study commenced in May 2024, and the first patients were enrolled in June 2024. Accrual is expected to be completed within 3 years. Informed consent will be documented, and patients will be given adequate time to consider participation in accordance with Clinical Trials Regulation (CTR) Article 29.

### Data collection and data management

Data will be recorded prospectively by local investigators using standardised electronic CRFs in Castor EDC, an ISO 27001-certified system. Patient-reported outcomes will be collected via electronic questionnaires, with paper versions available on request. The sponsor and investigator will retain the clinical trial master file for at least 25 years, per CTR Article 58.

### Data monitoring

Monitoring will be conducted in accordance with applicable laws, regulations and the Netherlands Federation of University Medical Centres (NFU) guideline for quality assurance of human research. This trial has been classified as moderate risk according to the NFU risk classification. No interim analysis is applicable to this trial.

### Recording and reporting of adverse events, serious adverse events and serious adverse reactions

The investigator will report all serious AEs and suspected unexpected serious adverse reactions (SUSARs) to the sponsor without undue delay on becoming aware of the event. The sponsor will submit an annual safety report to the Medical Ethical Board (METC) and competent authority throughout the clinical trial, in addition to expedited SUSAR reporting. The sponsor will maintain detailed records of all AEs reported by investigators (CTR Article 41(3)) and will promptly report relevant SUSARs to EudraVigilance. Fatal or life-threatening SUSARs will be reported within 7 days of first awareness. Non-fatal or non-life-threatening SUSARs will be reported within 15 days. If a SUSAR was initially considered non-fatal but is later classified as fatal, it will be reported within 7 days of the sponsor becoming aware.

### Auditing

Auditing will be conducted by independent qualified monitors at participating study centres. The study is classified as moderate risk according to the NFU guidelines outlined in Kwaliteitsborging Mensgebonden Onderzoek 2.0.

### Patient and public involvement

The Patiëntenadviesgroep (PAG), a collaboration between Borstkanker Onderzoek Groep (and Borstkankervereniging Nederland, along with Olijf, reviewed the research proposal and lay summary, providing feedback on relevance and outcome measures. PAG representatives also contributed to proofreading the patient information leaflet and study protocol. They are actively involved in the study, including the development of the implementation plan.

## Ethics and dissemination

### Research ethics approval

This study is approved by the Medical Ethical Board of Erasmus University Medical Centre MEC-2024-0030 and the institutional review boards of all participating centres.

### Protocol amendments

Any significant changes to the study protocol must receive approval from the central ethics committee. Once authorised, these modifications will be communicated to the Dutch competent authority, the institutional review boards of all study centres, all investigators, study registries and, if required, the patients.

### Informed consent

Informed consent will be obtained prior to any study-related procedures being undertaken at screening. Adequate time will be given for the subject to consider his or her decision to participate in the clinical trial (CTR: Article 29). The model consent form is provided in online supplemental file 1.

### Confidentiality

All personal data will be collected and processed in strict compliance with Dutch law and General Data Protection Regulation (EU 2016/679). Data will be pseudonymised, and access will be restricted to authorised study personnel and regulatory authorities.

### Access to data

The sponsor, principal investigators and designated researchers will have access to the final trial data set. No contractual agreements restrict data access.

### Ancillary or poststudy care

After the completion of trial participation, patients will continue standard-of-care treatment at the discretion of their treating physician, based on national clinical guidelines.

### Dissemination policy

Study results will be submitted for open-access publication in peer-reviewed medical journals and presented at national and international meetings for patients, healthcare professionals and the public. Positive outcomes will be locally implemented and integrated into routine clinical practice to improve patient care. Data from the clinical trial will be submitted to the EU portal Clinical Trials Information System, meeting the requirement for publicly accessible registration.

## Discussion

This randomised, multicentre trial investigates the complete omission of dexamethasone in premedication for paclitaxel-based chemotherapy. The primary objective is to assess the incidence of clinically relevant HSRs (CTCAE grade≥3). Secondary objectives include evaluating all-grade HSR incidence and severity, the number of paclitaxel administrations before the first HSR, dexamethasone-related AEs, patient-reported outcomes and cost-effectiveness.

Despite its routine inclusion since 1993, dexamethasone’s necessity for HSR prevention remains unproven as its use was extrapolated from radiocontrast hypersensitivity without direct comparative studies.^4 30^ Alternative premedication strategies, particularly omitting dexamethasone beyond cycle 2, have been explored.^12^,^16^ Retrospective data from Malmberg *et al* (personal correspondence) suggest that complete omission from the first cycle does not increase grade≥3 HSR incidence, yet prospective evidence is lacking, underscoring the need for this trial.

If dexamethasone proves unnecessary, its omission could reduce corticosteroid-related adverse effects such as hyperglycaemia, insomnia and immunodeficiency, improving HRQoL. Moreover, given the increasing use of paclitaxel in combination with immunotherapy, omitting dexamethasone may be clinically relevant as corticosteroids can potentially interfere with the efficacy of immunotherapy.^31^ Additionally, removing dexamethasone from premedication may lower healthcare and societal costs by reducing the management burden of corticosteroid-related AEs and eliminating unnecessary medication use.

This is the first randomised trial evaluating the safety and feasibility of complete dexamethasone omission in paclitaxel premedication. The findings could have broader implications for corticosteroid premedication strategies beyond paclitaxel, potentially guiding the optimisation of glucocorticosteroid use in premedication regimens for other chemotherapy agents or non-oncological treatments where corticosteroids are routinely used.

## Supplementary material

10.1136/bmjopen-2025-102770 online supplemental file 1
