# Supplementary material for: Omission of dexamethasone in paclitaxel premedication regimens: protocol of the multicentre, randomised, non-inferiority DEXASTOP trial
Source: BMJ Open. 2025 Apr 25;15(4):e102770. doi: 10.1136/bmjopen-2025-102770 (PMC12035437; doi:10.1136/bmjopen-2025-102770)
Supplement: online supplemental file 1 [file bmjopen-15-4-s001.docx]

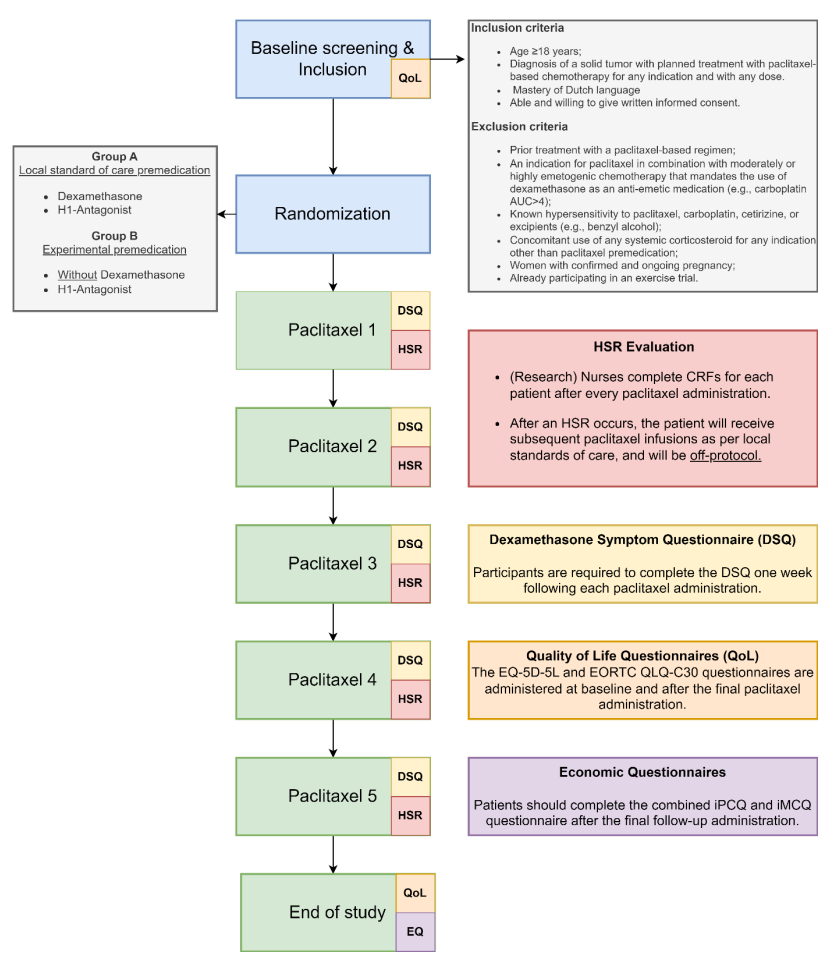


**Figure 1.** Study flowchart outlining patient enrollment, randomization, treatment allocation, follow-up assessments, and data collection points. HSR (hypersensitivity reaction), DSQ (Dexamethasone Symptom Questionnaire), QoL (quality of life), iPCQ (iMTA Productivity Cost Questionnaire), iMCQ (iMTA Medical Consumption Questionnaire).
